# Venous thromboembolism incidence and association with overall survival in pancreatic cancer: A Finnish nationwide cohort study

**DOI:** 10.1002/cam4.70014

**Published:** 2024-07-23

**Authors:** Panu Aaltonen, Harri Mustonen, Pauli Puolakkainen, Caj Haglund, Katriina Peltola, Olli Carpén, Riitta Lassila, Hanna Seppänen

**Affiliations:** ^1^ Department of Surgery, Translational Cancer Medicine Research Program, iCAN Digital Precision Cancer Medicine Flagship, Faculty of Medicine University of Helsinki and Helsinki University Hospital Helsinki Finland; ^2^ Comprehensive Cancer Center University of Helsinki and Helsinki University Hospital Helsinki Finland; ^3^ Medicum, Research Program in Systems Oncology and HUSLAB University of Helsinki Helsinki Finland; ^4^ Department of Hematology, Coagulation Disorders Unit and Research Program Unit in Systems Oncology (ONCOSYS), Faculty of Medicine University of Helsinki Helsinki Finland

**Keywords:** epidemiology, incidence, overall survival, pancreatic cancer, venous thromboembolism

## Abstract

**Introduction:**

Pancreatic cancer (PC) is associated with a high risk of venous thromboembolic events (VTEs). We investigated the incidence of VTE before and after the diagnosis of PC and its association with overall survival.

**Methods:**

We identified PC patients diagnosed in 2013–2016 from the Finnish Cancer Registry. Data on healthcare visits and death were collected, along with follow‐up data through the end of 2020. We compared patients who underwent radical‐intent surgery (RIS) to those who underwent palliative treatment (PT) alone.

**Results:**

We identified 4086 PC patients, of whom 343 (8.4%) underwent RIS and 3743 (91.6%) received PT. VTE incidence within 1 year before a PC diagnosis was higher in the PT (4.2%, *n* = 156) than in the RIS group (0.6%, *n* = 2; *p* < 0.001). The cumulative incidence of VTE at 12 and 24 months after a PC diagnosis was 6% (*n* = 21) and 9% (*n* = 31), respectively, within the RIS group, and 8% (*n* = 286) and 8% (*n* = 304) within the PT group. In the PT group, a VTE within 1 year before a PC diagnosis was independently associated with a worse survival {hazard ratio, HR 1.9 [95% confidence interval (CI) 1.6–2.2]}. In both groups, VTE after a PC diagnosis was associated with a worse survival [RIS group: HR 2.6 (95%CI 1.8–3.7) vs. PT group: HR 2.2 (95%CI 1.9–2.4)].

**Conclusions:**

A VTE within 1 year before a PC diagnosis more often occurred among PT PC patients than among patients who underwent RIS. VTE might serve as a diagnostic clue to detect PC at an earlier stage.

## Graphical abstract

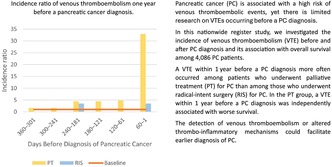

## 1 INTRODUCTION

Pancreatic cancer (PC) is one of the deadliest cancers, with annual incidence and mortality increasing.^1^ The risk of venous thromboembolism (VTE) associated with PC is well known and among the highest of all solid tumors.^2^, ^3^, ^4^ In PC patients, both symptomatic and asymptomatic VTEs are common.^2^, ^5^, ^6^, ^7^ Patients undergoing chemotherapy for metastasized PC are at an even greater risk of VTE.^4^, ^8^, ^9^, ^10^, ^11^ VTEs detected early following a PC diagnosis associate with a worse survival and are indicative of more aggressive tumor pathology.^2^, ^12^, ^13^, ^14^, ^15^

Among various common cancers, the incidence of an unprovoked VTE in the year preceding a cancer diagnosis is higher compared with the incidence in the general population.^16^ Furthermore, around 10% of individuals with an idiopathic VTE receive a cancer diagnosis within 1 year of the event.^16^, ^17^, ^18^ VTEs are also common preceding a PC diagnosis.^2^, ^9^, ^13^, ^16^ Blom et al. reported an incidence of deep vein thrombosis (DVT) in 6 of 1000 persons within a year preceding a PC diagnosis, while the population‐based incidence stood at 1–2 per 1000.^8^ In a French multicenter prospective study, 8% (91/1147) of newly diagnosed PC patients experienced a VTE at the time of enrolment or within the previous 3 months.

There is, however, a limited amount of existing literature specifically focused on VTEs that occur prior to a PC diagnosis. Thus, it remains unclear at what point in the progression of PC VTE incidence begins to increase and how often a VTE is indicative of occult PC. Moreover, it remains unclear whether the incidence of VTE preceding a PC diagnosis is prevalent among all patients or primarily observed among those with advanced disease. Additionally, there is a lack of knowledge regarding the impact of a VTE occurring before a PC diagnosis on overall survival (OS).

We, therefore, hypothesized that VTEs diagnosed prior to a PC diagnosis would associate with aggressive disease, and, thus, be more common in nonresectable cases and associate with a worse survival. This national register study aimed to investigate the incidence of VTE, the relationship between the temporal association of VTEs and establishing a PC diagnosis, and the impact on OS among PC patients in a population‐based nationwide cohort.

## 2 METHODS

The study protocol was approved by the National Institute for Health and Welfare (THL/1255/5.05.00/2018), Statistics Finland (TK‐52‐832‐19), and the Helsinki University Hospital (§91 HUS/419/2018).

From the Finnish Cancer Registry (FCR), we identified patients with PC (ICD‐10 code: C25) diagnosed between 2013 and 2016. Cause and time of death data were available until the end of 2020 and were collected from Statistics Finland. Healthcare visits from 2000 to 2018 were collected from the Care Register for Health Care (HILMO) from the National Institute for Health and Welfare. Data on primary healthcare outpatient visits were limited to the period 2011–2018. The register data include medical visit diagnoses and procedures, but do not include complete patient records. All VTEs, including ICD‐10 codes I80 [deep vein thrombosis (DVT)], I26 [pulmonary embolism (PE)], and IDC‐10 I81–82 [other venous thromboembolism (OVT)], occurring in 2000–2018 were identified. VTEs were categorized into three groups according to the time of occurrence: >1 year before a PC diagnosis, ≤1 year before a PC diagnosis, and after a PC diagnosis. We established a baseline for the incidence of VTE from the period 2–5 years prior to a PC diagnosis, considering the age‐related increase in VTE risk in the general population.^19^ If there were multiple visits related to a specific VTE diagnosis, only the first was included because it was not possible to distinguish whether these visits represent separate VTE events or if they are multiple visits related to the same initial VTE event.

Based on the Nordic Classification of Surgical Procedures, patients who underwent pancreatic surgery were identified from the HILMO data. Similarly, chemo‐ and radiation therapies were identified from the HILMO data and from a local register specific to the Helsinki and Uusimaa Hospital District (*n* = 491 patients). These datasets were, then, merged. By comparing the dates of oncological treatments and surgeries, we determined whether the oncological treatment was only preoperative, only postoperative, or perioperative for patients who underwent surgery. Information about specific chemotherapy agents was not available. Comorbidities according to the Charlson Comorbidity Index (CCI) were identified from visits preceding a PC diagnosis.^20^, ^21^ Disease stage information corresponding to the American Joint Committee on Cancer (AJCC)/Union for International Cancer Control (UICC) TNM staging system is not included in the FCR data. Preliminary analyses revealed inconsistencies and poor quality in the stage data reported in the registry, thus it was not used. We compared patients who underwent radical‐intent surgery (RIS) to those who underwent palliative treatment (PT) only. We calculated OS, censoring patients still living as of 31 December 2020. In 107 cases, the initiation of chemo‐ or radiation therapy preceded the date of diagnosis, and among 75 patients who underwent RIS, surgery was performed prior to the date of a PC diagnosis in the FCR.

We excluded patients younger than 18 years of age (*n* = 3) or who received a diagnosis of a neuroendocrine tumor (ICD‐10 code C25.4) or pancreatic tumor enucleation procedure (*n* = 189). We also excluded cases in which a PC diagnosis was assigned post‐mortem [based on death certificate (*n* = 172) or autopsy (*n* = 228)]. In addition, the following exclusion criteria relied on the histology report: neuroendocrine carcinoma (*n* = 27), carcinoid tumor (*n* = 154), solid pseudopapillary tumor (*n* = 3), and sarcoma (*n* = 3).

Statistical analyses were performed using IBM's SPSS Statistics for Windows, version 27.0.1 (IBM Corp, Armonk, NY, USA) and R, version 4.2.0 with survival package.^22^ The Fisher's exact test or the chi‐squared test were used to analyze categorical variables. The Mann–Whitney *U*‐test was used to compare continuous variables. Survival among different groups was analyzed using the Kaplan–Meier method, and the log‐rank test was used to determine statistical significance. We considered *p* < 0.05 statistically significant applying two‐tailed tests.

The Cox's proportional hazards model was used for univariable and multivariable analyses of prognostic factors for survival using VTEs after diagnosis and oncological treatments as time‐dependent variables. The Simon–Makuch method was used to create plots from survival data with the time‐dependent variables. Competing Cox models were used to evaluate the risk for VTE using overall death as a competing event. The Aalen–Johansen method was used to create plots from competing time to event data and to evaluate cumulative incidence functions. One patient who underwent chemotherapy for PC over a year before RIS was excluded from the Cox models. Furthermore, among RIS patients if oncological treatment was initiated more than 1 year after RIS, they were identified as having undergone no chemo‐ or radiation therapy in the multivariable model to address the effect of the primary treatment. An immortal time bias was corrected for oncological treatment using the time‐dependent variables to classify patients into treatment groups in a timely manner. The Cox regression assumption of a constant hazard ratio over time (proportional hazards) was assessed using the Schoenfeld residuals plotted over time and testing for a trend. A split time axis was used if necessary to account for deviances from the proportional hazard assumption. Interactions were considered.

## 3 RESULTS

### 3.1 Cohort characteristics

We identified 4086 PC patients, of whom 8% (*n* = 343) underwent RIS. Table 1 summarizes the patient characteristics. Patients in the PT group were older, with a median age of 74 [interquartile range (IQR) 66–81] years compared to 68 (IQR 62–74) years in the RIS group (*p* < 0.001). Twenty‐nine percent (*n* = 1086) of patients among the PT and 3% (*n* = 11) of patients among the RIS group were 80 years old or older.

**TABLE 1:** Patient characteristics.

Variable	All patients	Radical surgery	Palliative treatment	*p* ^a^
Total cases, *n* (%)	4086	343 (8)	3743 (92)	
Median age (IQR)	73 (66–81)	68 (62–74)	74 (66–81)	<0.001
Female, *n* (%)	2143 (52)	174 (51)	1969 (53)	0.534
Median follow‐up, in months (IQR)	4 (1–11)	27 (14–51)	3 (1–9)	<0.001
Charlson Comorbidity Index, *n* (%)				
0	1672 (41)	166 (48)	1506 (40)	0.002
1	926 (23)	78 (23)	848 (23)	
2	683 (17)	56 (16)	627 (17)	
≥ 3	805 (20)	43 (13)	762 (20)	
Histology				
Unknown	1939 (47)		1939 (52)	<0.001
Adenocarcinoma, NOS	1751 (43)	207 (60)	1544 (41)	
Ductal adenocarcinoma	294 (7)	120 (35)	174 (5)	
Mucinous adenocarcinoma	42 (1)	5 (1)	37 (1)	
Other	60 (1)	11 (3)	49 (1)	
Oncological treatment, *n* (%)				
Chemotherapy	1327 (33)	239 (70)	1088 (29)	<0.001
Radiotherapy	210 (5)	36 (11)	174 (5)	<0.001
Preoperative	55 (1)	55 (16)	NA	
Only postoperative	186 (5)	186 (54)	NA	

^a^ The *p*‐value compares the radical surgery group to the palliative treatment group.

Abbreviations: IQR, interquartile range; NA, not applicable; NOS, not otherwise specified.

Additionally, the PT group had a higher prevalence of chronic illnesses. Specifically, 20% of patients in the PT group had a CCI score ≥3, compared with 13% in the RIS group (*p* = 0.002). Furthermore, fewer patients in the PT group had a CCI score of 0 than the proportion in the RIS group (40% vs. 48%). Table S1 provides the distribution of CCI comorbidities among treatment groups. In total, 13.5% (*n* = 550) of all patients had a history of other malignancies. More specifically, prostate cancer (*n* = 139), breast cancer (*n* = 132), colorectal cancer (*n* = 80), endometrial cancer (*n* = 32), and melanoma (*n* = 31) stood out as the most frequent other malignancies. In addition, 18.8% (*n* = 770) of patients had a history of atrial fibrillation, which was more common in the PT group (19.3%, *n* = 723) than the RIS group (13.7%, 0 = 47; *p* = 0.011).

RIS consisted of a pancreatoduodenectomy in 280 (82%) patients, a distal pancreatectomy in 37 (11%) patients, a total pancreatoduodenectomy in 22 (6%) patients, and other pancreatic resection in 4 (1%) patients.

### 3.2 Venous thromboembolism prior to a PC diagnosis

**FIGURE 1:**
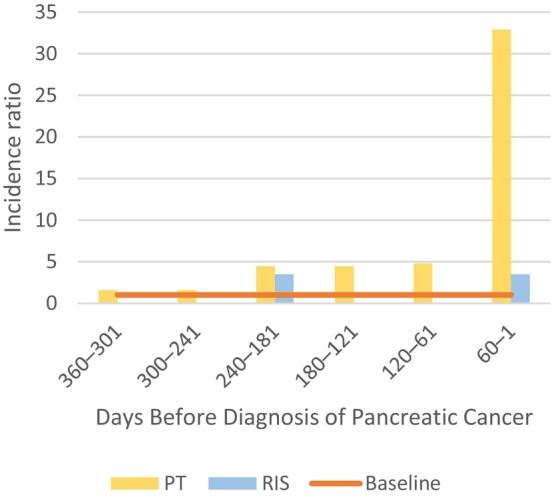
Incidence of venous thromboembolism events 1 year before a pancreatic cancer diagnosis in two‐month increments. PT, palliative treatment; RIS, radical‐intent surgery; baseline, average incidence of VTE in the 2–5 years before a pancreatic cancer diagnosis (502 per 100,000 annually) in the cohort. The incidence ratio was calculated comparing the baseline incidence to the observed incidence among groups.

Table 2 captures all VTEs in the study population, stratified by the timing relative to a PC diagnosis. Considering VTEs that occurred >1 year before a PC diagnosis, there were no differences between the groups given that 5% (*n* = 16) of patients in the RIS group and 4% (*n* = 165) of patients in the PT group experienced a VTE >1 year before a PC diagnosis (*p* = 0.825). The annual incidence of VTE at 2, 3, 4, and 5 years before a PC diagnosis was 534, 508, 508, and 481 per 100,000, respectively, in the PT group, and 292, 583, 292, and 583 per 100,000, respectively, in the RIS group (*p* = 0.666). The average annual incidence of VTE in the period 2–5 years prior to a PC diagnosis stood at 502 per 100,000. The incidence ratio of VTE in two‐month increments in the year before a PC diagnosis appears in Figure 1.

**TABLE 2:** Venous thromboembolisms among radical‐intent surgery and palliative treatment patients before and after the diagnosis of pancreatic cancer.

Venous thromboembolism (VTE)^a^	All patients (*n* = 4086)	Radical‐intent surgery (*n* = 343)	Palliative treatment (*n* = 3743)	*p* ^b^
	No. of VTE (%)	No. of VTE (%)	No. of VTE (%)	
>1 year before PC diagnosis	181 (4.4)	16 (4.7)	165 (4.4)	0.825
≤1 year before PC diagnosis	158 (3.9)	2 (0.6)	156 (4.2)	<0.001
After PC diagnosis	367 (9.0)	42 (12.2)	325 (8.7)	0.027

^a^ Any venous thromboembolism including deep venous thrombosis, pulmonary embolism, or visceral vein thromboembolism.

^b^ Radical‐intent surgery versus palliative treatment.

Abbreviations: No, number; PC, pancreatic cancer.

In the PT group, a larger proportion of patients experienced a VTE ≤1 year before a cancer diagnosis, consisting of 0.6% (95% CI 0.1%–2.1%) in the RIS group and 4.2% (95% CI 3.6%–4.9%) in the PT group (*p* < 0.001). More specifically, ≤1 year before a PC diagnosis, there were 78 PEs, 78 DVTs, and 28 OVTs in the PT group, while there was only 1 PE and 1 DVT in the RIS group. To further explore this, ≤1 year before PC diagnosis VTEs were stratified by age groups in Table S2. A logistic regression investigating the odds of venous thromboembolism within 1 year before the diagnosis of PC is presented in Table S3. While CCI sum three or over and age were independent risk factors, RIS group (odds ratio 0.18) was an independent factor for lower VTE risk. In Table S1, we compared comorbidities between the PT and RIS groups, and the only significant differences were in congestive heart failure and cerebrovascular disease.

### 3.3 Venous thromboembolism after a PC diagnosis

**FIGURE 2:**
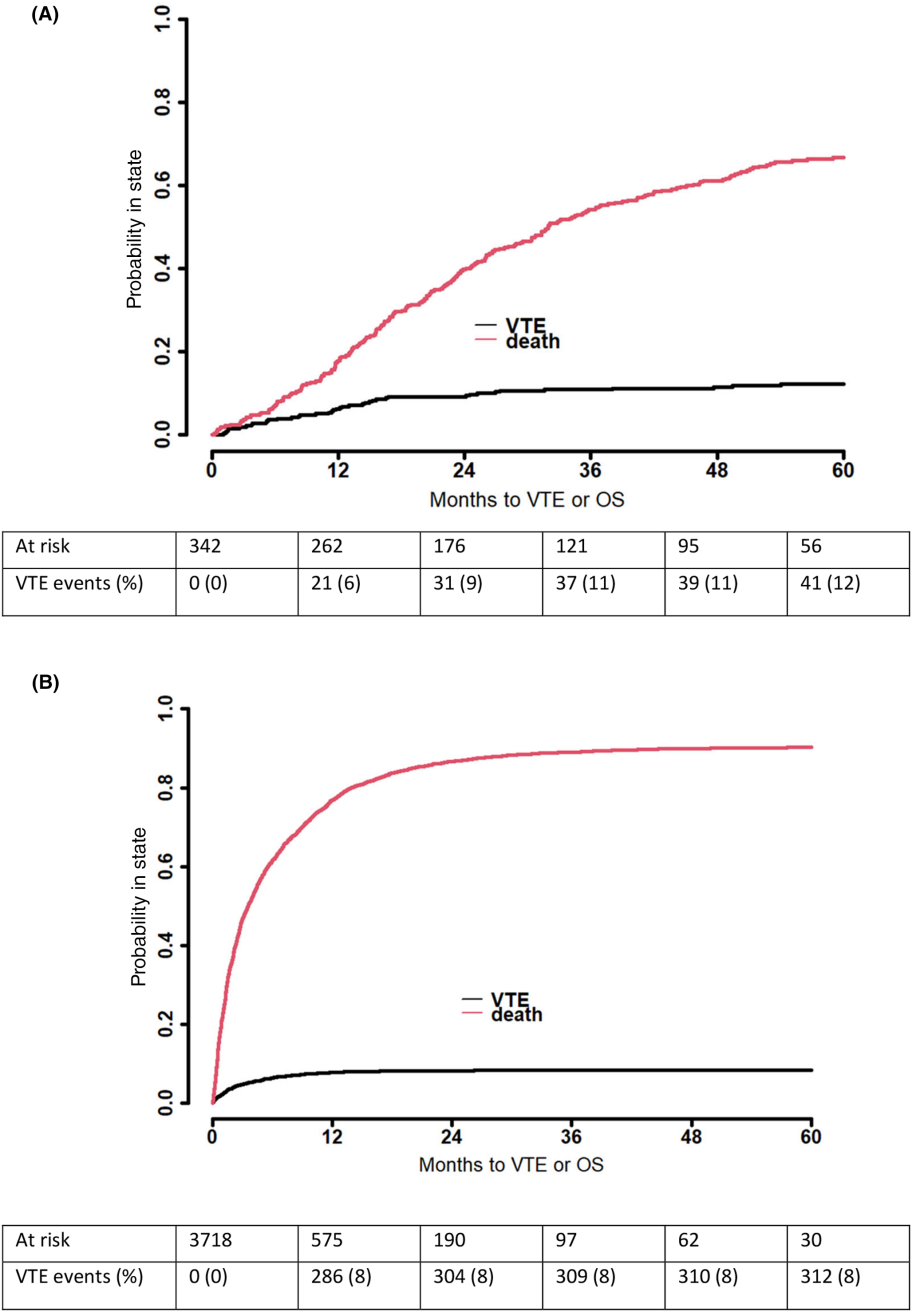
Cumulative incidence function of venous thromboembolism among (A) radical surgery (*n* = 342 patients) and (B) palliative treatment (*n* = 3718 patients) groups after a pancreatic cancer diagnosis. Death analyzed as a competing factor. OS, overall survival; VTE, venous thromboembolism.

A total of 367 (9%) patients experienced a VTE after a PC diagnosis. In total, there were 193 PEs, 143 DVTs, and 77 OVTs following a PC diagnosis. In addition, 42 (12%) patients in the RIS and 325 (9%) patients in the PT group experienced a VTE after a PC diagnosis. The cumulative incidence of VTE at 12 and 24 months after a PC diagnosis was 6% (*n* = 21) and 9% (*n* = 31), respectively, among the RIS group, and 8% (*n* = 286) and 8% (*n* = 304), respectively, in the PT group. The median time from a PC diagnosis until a VTE was 65 (IQR 21–170) days among the PT group and 355 (IQR 153–566) days among the RIS group. Figure 2 illustrates the cumulative incidence function of VTE following a PC diagnosis stratified by the RIS and PT groups.

Among the RIS group, the cumulative VTE incidence at 3, 12, and 24 months was 1.0% (*n* = 1), 5.5% (*n* = 3), and 7.3% (*n* = 4) for those receiving neoadjuvant treatment; 1.1% (*n* = 2), 5.9% (*n* = 11), and 9.1% (*n* = 17) for those receiving only adjuvant treatment; and 2.9% (*n* = 3), 7.8% (*n* = 8), and 10.8% (*n* = 11) for those receiving no chemo‐ or radiation therapy in addition to surgery (*p* = 0.923). The cumulative VTE incidence at 3 months after RIS was 2.0% (*n* = 8).

Among the PT group, the cumulative VTE incidence at 3, 12, and 24 months was 6.9% (*n* = 115), 13.2% (*n* = 160), and 14.6% (*n* = 164) for those receiving chemotherapy and 4.4% (*n* = 73), 6.1% (*n* = 139), and 6.2% (*n* = 154) for those not receiving chemotherapy (*p* < 0.001).

### 3.4 Venous thromboembolism as cause of death

When we investigated the underlying and immediate causes of death as well as any contributing conditions recorded, we found that VTE was not defined as the underlying cause of death for any of the patients in this series. However, VTE was listed as an immediate cause of death in 22 (0.5%) cases and a contributing condition in 83 (2.0%) cases. PE was recorded as the immediate cause of death in 22 (0.5%) patients and a contributing factor in another 71 patients (1.7%). DVT was a contributing factor in 15 (0.4%) cases, while OVT was a contributing factor in 2 (0.05%) cases. Results S4 provides the cause of death among cases excluded due to the post‐mortem report.

### 3.5 Survival and predictors

Among all patients, median OS was 3.7 months (95% CI 3.5–4.0), the 1‐year survival rate was 22%, the 3‐year survival rate was 5.7%, and the 5‐year survival rate was 3.1%. Median OS was 27 months (95% CI 23–31) among patients in the RIS group and 3.1 months (95% CI 2.9–3.3) among those in the PT group (*p* < 0.001). The respective 1‐, 3‐, and 5‐year survival rates were 79%, 38%, and 22% among the RIS group, and 16%, 2.9%, and 1.3% among the PT group.

The multivariate model (Table 3) revealed that VTE following a PC diagnosis represented a significant prognostic factor for a worse OS (HR 2.11 [95%CI 1.88–2.38], *p* < 0.001 among the RIS group; HR 2.57 [95%CI 1.79–3.69], *p* < 0.001 among the PT group). However, neither a VTE >1 year before a PC diagnosis (HR 0.87 [95%CI 0.47–1.60], *p* = 0.655) nor ≤1 year before a PC diagnosis (HR 1.90 [95%CI 0.59–6.15], *p* = 0.283) associated with survival in the RIS group. However, we identified only two patients who experienced a VTE ≤1 year before a PC diagnosis in this group. By contrast, in the PT group, a VTE ≤1 year prior to a PC diagnosis associated with a worse survival (HR 1.86 [95%CI 1.58–2.19], *p* < 0.001). A higher age (HR 1.03 [95%CI 1.02–1.03], *p* < 0.001) and a CCI score ≥3 (HR 1.24 [95%CI 1.13–1.36], *p* < 0.001) also associated with a higher risk of death. Female sex (HR 0.90 [95%CI 0.84–0.96], *p* = 0.002) associated with a better survival. In both groups, oncological therapies associated with a lower risk of death during the first year of follow‐up, but this effect was subsequently nullified.

**TABLE 3:** Multivariable analysis of the risk of death among pancreatic cancer patients considering venous thromboembolic events at three different time intervals: Over 1 year before, within 1 year before, and following a pancreatic cancer diagnosis. (A) Radical‐intent surgery (*n* = 342). (B) Palliative treatment (*n* = 3718)^a^.

Multivariable	HR for OS death	95% CI Lower	95% CI Upper	*p*
**(A) Radical‐intent surgery**				
Age	1.002	0.987	1.017	0.775
Sex (female)^b^	0.888	0.698	1.131	0.336
CCI 1	0.951	0.697	1.296	0.748
CCI 2	1.029	0.721	1.469	0.876
CCI 3+	1.099	0.751	1.608	0.628
VTE >1 year before PC dg	0.870	0.472	1.604	0.655
VTE ≤1 year before PC dg	1.902	0.589	6.145	0.283
VTE after PC dg	2.571	1.791	3.691	<0.001
Chemo‐ and/or radiation therapy^c^				
0–12 months	0.491	0.295	0.818	0.006
>12 months	1.042	0.7622	1.423	0.798
**(B) Palliative treatment**				
Age	1.026	1.023	1.030	<0.001
Sex (female)^b^	0.900	0.843	0.962	0.002
CCI 1	1.038	0.953	1.131	0.397
CCI 2	1.028	0.934	1.131	0.572
CCI 3+	1.239	1.130	1.358	<0.001
VTE >1 year before PC dg	1.061	0.905	1.242	0.467
VTE ≤1 year before PC dg	1.857	1.576	2.188	<0.001
VTE after PC dg	2.111	1.875	2.377	<0.001
Chemo‐ and/or radiation therapy^c^				
0–12 months	0.633	0.578	0.693	<0.001
>12 months	0.863	0.726	1.025	0.092

^a^ One radical‐intent surgery patient who underwent oncological treatment for pancreatic cancer over 1 year before the radical‐intent surgery was excluded from the model. In addition, radical surgery patients who underwent oncological treatment only over 1 year after surgery were considered no chemotherapy or no radiation therapy in this model. Patients who had died at the start of the follow‐up period were excluded from this model (*n* = 25 among the palliative treatment group).

^b^ A minor deviance from the proportional hazard assumption for sex was ignored.

^c^ The time axis was split to account for deviances from the proportional hazard assumption. Chemo‐ and/or radiation therapy and VTE following a pancreatic cancer diagnosis represent time‐dependent variables.

Abbreviations: CCI, Charlson Comorbidity Index; CI, confidence interval; dg, diagnosis; HR, hazard ratio; OS, overall survival; PC, pancreatic cancer; VTE, venous thromboembolism.

**FIGURE 3:**
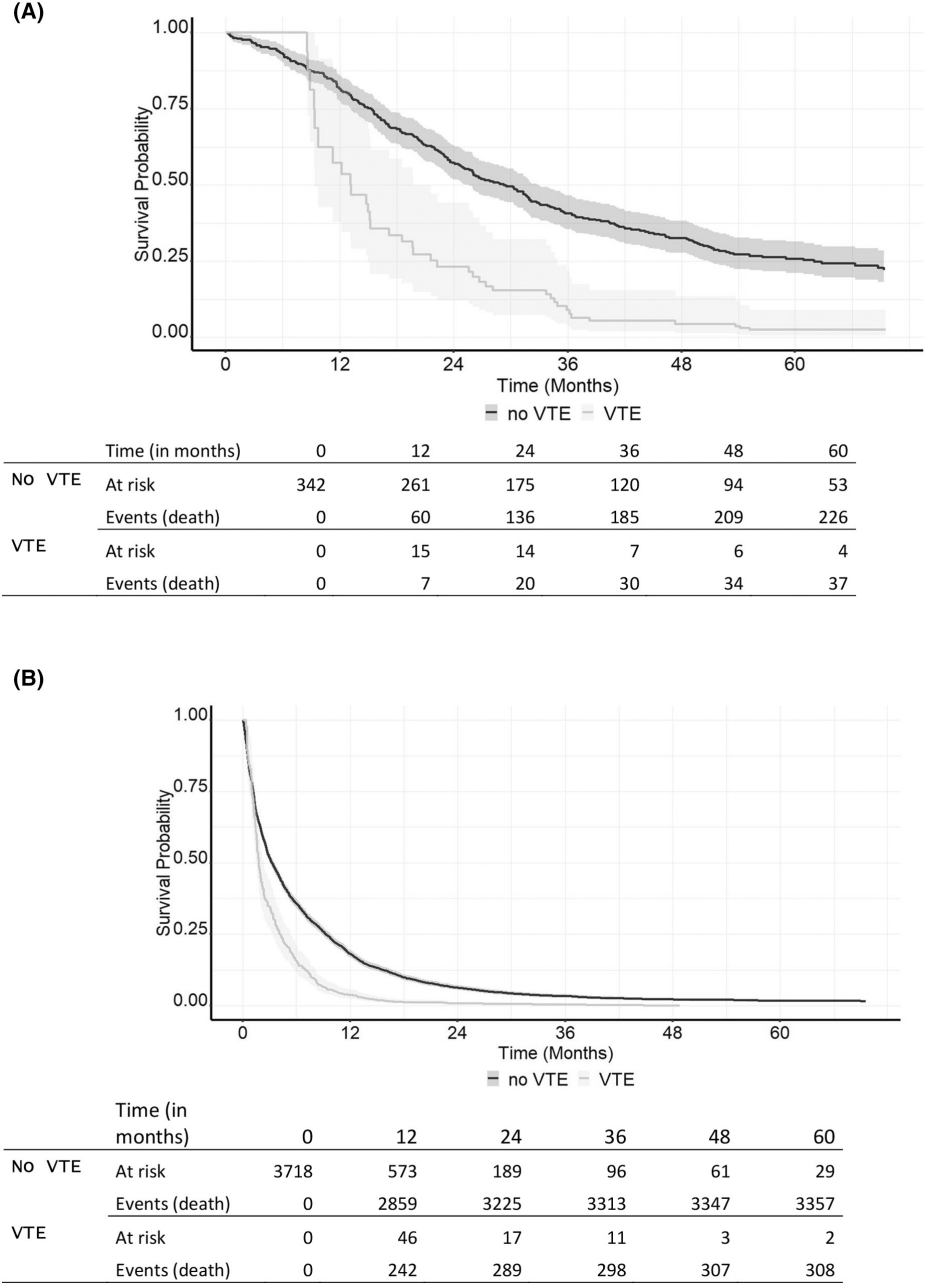
The Simon–Makuch figures illustrating the impact of venous thromboembolism after a diagnosis of pancreatic cancer on overall survival among (A) radical‐intent surgery (*n* = 342) and (B) palliative treatment (*n* = 3718) patients. VTE was used as a time‐dependent variable.

The Simon–Makuch analysis in Figure 3 illustrates the impact of VTE after a PC diagnosis on OS among the RIS group and the PT group. VTE associated with a significantly worse survival in both groups (Figure 3, Table 3).

## 4 DISCUSSION

In this nationwide, population‐based, cohort study, we found that patients who underwent PT for PC had a significantly higher VTE incidence (4.2%) in the year before a PC diagnosis compared with the RIS group (0.6%). These VTEs significantly affected prognosis in the PT group (HR 1.9 for death), but were not a significant factor in the RIS group, although the limited number of observations (*n* = 2) in this group reduced the statistical power in the survival analysis. VTE incidence among PC patients was elevated as early as 8 months before the diagnosis, indicating a potential association with an occult PC.

Only a limited number of studies have addressed the increased VTE incidence preceding a PC diagnosis. A California Cancer Registry–based study observed remarkably lower rates than in our study. In their data, 0.2% (*n* = 34/13731) of PC patients had an unprovoked VTE within a year prior to diagnosis.^16^ They found that incidence was significantly elevated in the 4 months immediately preceding a cancer diagnosis and that VTEs associated with metastatic disease. In another register study from the United States, the incidence of venous and arterial thromboembolisms among cancer patients was highest between 90 days before and 240 days after a cancer diagnosis, peaking at 14 days following a diagnosis.^23^ In a study by Riedl et al. among patients with advanced PC, 13% had a cancer‐associated VTE prior to the initiation of first‐line palliative chemotherapy, while 5% had a previous medical history of VTE.^24^ In our study, the baseline incidence of VTE (502 per 100,000) we observed in the 2–5 years before a PC diagnosis corresponds with the reported incidence in the general population at a similar age range as that from a US register study, which reported an incidence of 480 per 100,000 among individuals aged 70–74.^25^

Our data revealed that 9% of patients had a VTE following a PC diagnosis. A British register study of 67,801 cancer patients indicated that cancer‐associated VTE occurred in 2.2% of patients, while the VTE rate among PC patients was 4.7% (*n* = 47/999).^4^ A Japanese nationwide multicenter observational study reported a VTE incide of 8.5% among stage II–IV PC patients with planned cancer treatment.^6^ In their data, VTE prevalence was lower in other cancer types: 6.9% in stomach cancer, 6.4% in colorectal cancer, 5.5% in gynecologic cancer, 5.1% in lung cancer, and 2.0% in breast cancer. Furthermore, 1‐year cumulative VTE incidence was 3.6% among PC patients compared to 1.6% among other cancers. In a recent retrospective Finnish single‐center study from Turku University Hospital, the rate of VTE among PC patients in that catchment population during 2005–2013 was 5.5%.^11^ In the French, prospective, multicenter study among newly diagnosed PC patients at all stages of disease, at 6 months and 1 year, respectively, the cumulative rates of VTE were 13% and 20%.^2^ In a Korean population‐based study, 2‐year cumulative VTE incidence among PC patients was 9.2%.^5^ Reported VTE rates among PC patients vary widely for many reasons, including differences in the study population, follow‐up, the definition of VTE, diagnostic techniques as well as data source integrity.^26^ Moreover, it is essential to acknowledge the statistical method used to estimate cumulative incidence.^27^ Furthermore, a recent review and meta‐analysis concluded that the study objectives may impact the incidence of VTE given that the rate of VTEs in PC chemotherapy trials is lower than in thromboprophylaxis trials.^28^ The pooled rate of PC‐associated VTE in chemotherapy studies was 5.9%, climbing to 16.5% in thromboprophylaxis studies.

Among patients undergoing RIS, both surgery and chemotherapy were previously reported risk factors for VTE.^29^, ^30^ Thus, the perioperative period is of special interest. In our data, postoperative VTE incidence at 3 months following surgery stood at 2.0%. A recent meta‐analysis reported that the estimated four‐week postoperative risk of symptomatic VTE without thromboprophylaxis was 6.2% following an open pancreatoduodenectomy for benign and malignant indications; yet, that estimate was deemed to have a low level of certainty.^29^ A US prospective single‐center study found that among pancreatic adenocarcinoma patients undergoing either curative or palliative surgery exhibited a 12% postoperative cumulative incidence of VTE at 3 months following surgery despite thromboprophylaxis lasting for 21 days post‐operatively.^30^ While the specific stage distribution was not reported, 9% had metastatic disease and the pre‐operative chemotherapy rate of 14.5% was similar to our data.

The role of neoadjuvant treatment on the risk of VTE has received increasing attention in recent years. In our data, we found no statistical difference in the VTE incidence among RIS patients undergoing neoadjuvant or adjuvant therapy alone. In a single‐center prospective US study, 10% of patients undergoing neoadjuvant treatment for resectable or borderline resectable PC experienced a VTE during neoadjuvant chemotherapy.^31^ VTE associated with the impaired completion of therapy as well as a worse OS. In a recent Finnish retrospective single‐center study, neoadjuvant treatment was identified as an independent VTE risk factor.^32^ In their study, neoadjuvant treatment associated with an increased VTE incidence up to 2 years following surgery, and a HR of 1.61 for VTE compared with upfront surgery. Furthermore, a majority of VTEs (*n* = 58/87) followed disease recurrence and VTE was infrequent among patients who had no disease recurrence. Similarly, we found that a significant proportion of RIS patients developed a VTE as time passed. This can be reasonably attributed to the recurrence of PC, as reflected by the poor prognosis among patients. Similar to other studies, PC recurrence has been associated with an increased VTE risk.^24^

Among the PT group, VTEs within 1 year before a PC diagnosis were prevalent and associated with a 1.9‐fold risk of death. Furthermore, the absence of these VTEs among the RIS group casts doubt on whether VTE may have facilitated the earlier detection of PC among the PT group, resulting in improved survival outcomes. VTE has been reported to be associated with poorer overall and disease‐specific survival among advanced PC patients, especially if VTE is detected early after diagnosis.^7^, ^15^ In our data, the occurrence of VTE following a PC diagnosis associated with a HR of 2.1 for death among the PT group. However, VTE was rarely the cause of death. In agreement with the findings of Riedl et al., a diminished survival cannot be attributed to direct VTE‐related effects, but instead to an aggressive cancer pathology coupled with a hypercoagulable state.^24^ The presence of metastatic disease is a major risk factor for VTE in patients with PC.^8^ In several retrospective cohort studies, the incidence of VTE among advanced PC patients undergoing palliative chemotherapy was high, ranging from 20% to 26%.^9^, ^12^, ^24^, ^33^ Patients diagnosed with VTE around the initiation of chemotherapy appear to experience a worse prognosis as well as a poor response to chemotherapy.^12^, ^33^ However, in a recent singe‐center retrospective Japanese study, unresectable metastatic pancreatic adenocarcinoma patients with VTE had similar response rates for first‐line gemcitabine plus nab‐paclitaxel combination chemotherapy, but the rate of second‐line chemotherapy was lower in the VTE group.^7^

The abysmal prognosis of advanced PC highlights the crucial need for early detection methods. The association between new‐onset diabetes mellitus and PC is well recognized, however, the absolute risk of PC among new‐onset diabetes patients is low (0.85% during a three‐year follow‐up).^34^, ^35^, ^36^ A model based on weight loss, blood glucose, and age at the onset of diabetes has been suggested to identify new‐onset diabetic individuals at risk of developing PC.^37^ Incorporating of venous thromboembolism in a screening tool has not been reported. The detection of altered thrombo‐inflammatory mechanisms might contribute to the diagnosis of early‐stage PC and be used in combination with other factors to create a feasible and cost‐effective screening protocol.^38^, ^39^, ^40^, ^41^, ^42^, ^43^

We observed a high prevalence of comorbidities among both PT and RIS patient groups. The most notable among these were cardiovascular diseases, chronic pulmonary disease, diabetes, and a history of other malignancies. Interestingly, none of the comorbidities analyzed were significantly more prevalent among patients with a PC‐associated VTE (Table S1). In the literature, obesity and cardiovascular diseases, encompassing atrial fibrillation/flutter, hypertension, cerebrovascular disease, and congestive heart failure, have been documented as factors associated with VTEs among hospitalized cancer patients.^11^

The strengths of this study are the large nationwide cohort and the high quality of nationwide Finnish registers.^44^, ^45^ The limitations include the secondary nature of register data more generally. The use of anticoagulation or other medications is not included. In addition, CCI addresses only some comorbidities. Moreover, the register data may not provide a comprehensive overview of a patient's chronic conditions. While a histology was obtained for all patients who underwent RIS, the report remained unknown in a substantial proportion of PT patients. The Finnish Cancer Register does not include cancer staging corresponding to the AJCC/UICC TNM staging. Thus, surgical treatment was selected to categorize prognostic groups. There is a possibility of missing data on VTEs since diagnosing and documentation of VTEs in clinical settings might not be as efficient as desired. Furthermore, the rate at which patients underwent oncological treatments remained low. A possible explanation lies in the incomplete recording of subsidiary diagnoses and secondary operations, especially related to outpatient visits.^44^ Additionally, it was impossible to determine whether VTEs were incidental or symptomatic. Clinical and laboratory variables necessary for calculating the Khorana score were unavailable.^3^ Also, other laboratory data, such as carbohydrate antigen 19–9 or D‐dimer levels, were unavailable.

VTE manifests as a frequent comorbidity among PC patients, bearing remarkable prognostic significance. VTE may provide an important diagnostic hint for detecting PC at an earlier stage. A better understanding of the pathways and biomarkers associated with an increased VTE risk may lead to earlier diagnosis and the development of new therapies for PC.

## AUTHOR CONTRIBUTIONS

**Panu Aaltonen:** Data curation (lead); formal analysis (lead); writing – original draft (lead). **Harri Mustonen:** Formal analysis (equal); writing – review and editing (equal). **Pauli Puolakkainen:** Conceptualization (equal); project administration (equal); supervision (equal); writing – review and editing (equal). **Caj Haglund:** Conceptualization (equal); writing – review and editing (equal). **Katriina Peltola:** Resources (equal); writing – review and editing (equal). **Olli Carpén:** Writing – review and editing (equal). **Riitta Lassila:** Methodology (equal); writing – review and editing (equal). **Hanna Seppänen:** Conceptualization (equal); funding acquisition (equal); methodology (equal); project administration (equal); supervision (equal); writing – review and editing (equal).

## FUNDING INFORMATION

This study was supported by the Helsinki University Hospital Research Fund and the Orion Research Foundation sr.

## CONFLICT OF INTEREST STATEMENT

The authors declare no conflicts of interest.

## ETHICS STATEMENT

The study protocol was approved by the National Institute for Health and Welfare (THL/1255/5.05.00/2018), Statistics Finland (TK‐52‐832‐19), and the Helsinki University Hospital (§91 HUS/419/2018). Due to the observational nature of the study and in accordance with local standards, neither written consent from patients nor an ethical committee statement was acquired.

## Supporting information


Data S1.

## Data Availability

Due to legal restrictions, the data underlying this study cannot be shared.
