# Supplementary material for: Venous thromboembolism incidence and association with overall survival in pancreatic cancer: A Finnish nationwide cohort study
Source: Cancer Med. 2024 Jul 23;13(14):e70014. doi: 10.1002/cam4.70014 (PMC11263919; doi:10.1002/cam4.70014)
Supplement: Supplementary file 1 — Data S1. [file CAM4-13-e70014-s001.docx]

Supplementary Table S1. Comorbidities according to the Charlson comorbidity index among pancreatic cancer patients stratified by treatment groups (A) and by any venous thromboembolism within one year before or after a pancreatic cancer diagnosis (B)

**A**

| Comorbidity | Radical-intent surgery  (n = 343) | | Palliative treatment (n = 3743) | |  | Total (n = 4086) | | | |
| --- | --- | --- | --- | --- | --- | --- | --- | --- | --- |
|  | n | (%) | n | (%) | *p** | n | | (%) |  |
| Myocardial infarction | 18 | (5.2) | 325 | (8.1) | 1.000 | 321 | (7.9) | | |
| Congestive heart failure | 11 | (3.2) | 332 | (10.3) | <0.001 | 395 | (9.7) | | |
| Peripheral vascular disease | 16 | (4.7) | 230 | (6.1) | 1.000 | 246 | (6) | | |
| Cerebrovascular disease | 27 | (7.9) | 551 | (14.7) | 0.004 | 578 | (14.1) | | |
| Dementia | 29 | (8.5) | 509 | (13.6) | 1.000 | 538 | (13.2) | | |
| Chronic pulmonary disease | 44 | (12.8) | 461 | (12.3) | 1.000 | 505 | (12.4) | | |
| Rheumatologic disease | 7 | (2.0) | 163 | (4.4) | 1.000 | 170 | (4.2) | | |
| Peptic ulcer disease | 7 | (2.0) | 115 | (3.1) | 1.000 | 122 | (3.0) | | |
| Mild liver disease | 6 | (1.7) | 89 | (2.4) | 1.000 | 95 | (2.3) | | |
| Diabetes without a chronic complication | 65 | (19.0) | 730 | (19.5) | 1.000 | 795 | (19.5) | | |
| Diabetes with a chronic complication | 7 | (2.0) | 130 | (3.5) | 1.000 | 137 | (3.4) | | |
| Hemiplegia or paraplegia | 2 | (0.6) | 19 | (0.5) | 1.000 | 21 | (0.5) | | |
| Renal disease | 1 | (0.3) | 80 | (2.1) | 0.212 | 81 | (2.0) | | |
| History of malignancy other than PC | 44 | (12.8) | 506 | (13.5) | 1.000 | 550 | (13.5) | | |
| Moderate or severe liver disease | 2 | (0.6) | 28 | (0.7) | 1.000 | 30 | (0.7) | | |
| Metastatic solid tumor other than PC | 2 | (0.6) | 28 | (0.7) | 1.000 | 30 | (0.7) | | |
| HIV/AIDS | 0 | (0) | 1 | (0.03) | 1.000 | 1 | (0.02) | | |

**B**

|  |  | | | | | | | | |  |  |  |
| --- | --- | --- | --- | --- | --- | --- | --- | --- | --- | --- | --- | --- |
| Comorbidity | Radical-intent surgery (n = 343) | | | |  | Palliative treatment (n = 3743) | | | |  | Total (n = 4086) | |
|  | VTE | | no VTE | |  | VTE | | no VTE | |  |  |  |
|  | n | (%) | n | (%) | *p** | n | (%) | n | (%) | *p*** | n | (%) |
| Myocardial infarction | 1 | (2.3) | 17 | (5.7) | 1.000 | 29 | (8.4) | 274 | (6.1) | 0.850 | 321 | (7.9) |
| Congestive heart failure | 2 | (4.7) | 9 | (3.0) | 1.000 | 27 | (5.7) | 357 | (10.9) | 0.002 | 395 | (9.7) |
| Peripheral vascular disease | 0 | (0) | 16 | (5.3) | 1.000 | 20 | (4.2) | 210 | (6.4) | 0.561 | 246 | (6) |
| Cerebrovascular disease | 2 | (4.7) | 25 | (8.3) | 1.000 | 65 | (13.7) | 486 | (14.9) | 1.000 | 578 | (14.1) |
| Dementia | 4 | (9.3) | 25 | (8.3) | 1.000 | 51 | (10.7) | 458 | (14.0) | 0.476 | 538 | (13.2) |
| Chronic pulmonary disease | 4 | (9.3) | 40 | (13.3) | 1.000 | 65 | (13.7) | 396 | (12.1) | 1.000 | 505 | (12.4) |
| Rheumatologic disease | 0 | (0) | 7 | (2.3) | 1.000 | 15 | (3.2) | 148 | (4.5) | 1.000 | 170 | (4.2) |
| Peptic ulcer disease | 2 | (4.7) | 5 | (1.7) | 1.000 | 12 | (2.5) | 103 | (3.2) | 1.000 | 122 | (3.0) |
| Mild liver disease | 1 | (2.3) | 5 | (1.7) | 1.000 | 11 | (2.3) | 78 | (2.4) | 1.000 | 95 | (2.3) |
| Diabetes without a chronic complication | 7 | (16.3) | 58 | (19.3) | 1.000 | 72 | (15.2) | 658 | (20.1) | 0.085 | 795 | (19.5) |
| Diabetes with a chronic complication | 0 | (0) | 7 | (2.3) | 1.000 | 14 | (2.9) | 116 | (3.5) | 1.000 | 137 | (3.4) |
| Hemiplegia or paraplegia | 0 | (0) | 2 | (0.7) | 1.000 | 2 | (0.4) | 17 | (0.5) | 1.000 | 21 | (0.5) |
| Renal disease | 1 | (2.3) | 0 | (0) | 1.000 | 9 | (1.9) | 71 | (2.2) | 1.000 | 81 | (2.0) |
| History of malignancy other than PC | 4 | (9.3) | 40 | (13.3) | 1.000 | 66 | (13.9) | 440 | (13.5) | 1.000 | 550 | (13.5) |
| Moderate or severe liver disease | 0 | (0) | 2 | (0.7) | 1.000 | 1 | (0.2) | 27 | (0.8) | 1.000 | 30 | (0.7) |
| Metastatic solid tumor other than PC | 1 | (2.3) | 1 | (0.3) | 1.000 | 7 | (1.5) | 21 | (0.6) | 1.000 | 30 | (0.7) |
| HIV/AIDS | 0 | (0) | 0 | (0) |  | 1 | (0.2) | 0 | (0) | 1.000 | 1 | (0.02) |

**Abbreviations:** VTE, any venous thromboembolism; PC, pancreatic cancer.

* Bonfferoni adjusted *p*-value for radical-intent surgery vs palliative treatment

**Bonfferoni adjusted *p*-value for VTE vs no VTE comparison.

Supplementary Table S2. Venous thromboembolism among radical surgery and palliative treatment patients before and after the diagnosis of pancreatic cancer stratified by age groups

|  | Radical-intent surgery (n = 343) | | | | | |  | Palliative Treatment (n = 3743) | | | | | |  |
| --- | --- | --- | --- | --- | --- | --- | --- | --- | --- | --- | --- | --- | --- | --- |
|  | Age | | | | | |  | Age | | | | | |  |
|  | ≤65 | | 66–79 | | ≥80 | |  | ≤65 | | 66–79 | | ≥80 | |  |
|  | *n*=136 | | *n*=196 | | *n*=11 | |  | *n*=907 | | *n*=1750 | | *n*=1086 | |  |
| VTE | *n* | (%) | *n* | (%) | *n* | (%) | *p** | *n* | (%) | *n* | (%) | *n* | (%) | *p** |
| >1 y before PC Diagnosis | 6 | (4.4) | 9 | (4.6) | 1 | (9.1) | 0.696 | 21 | (2.3) | 69 | (3.9) | 75 | (6.9) | <0.001 |
| ≤1 y before PC Diagnosis | 2 | (1.5) | 0 | (0) | 0 | (0) | 0.098 | 50 | (5.5) | 77 | (4.4) | 29 | (2.7) | 0.001 |
| After PC Diagnosis | 22 | (16.2) | 20 | (10.2) | 0 | 0 | 0.043 | 121 | (13.3) | 170 | (9.7) | 34 | (3.1) | <0.001 |
|  |  |  |  |  |  |  |  |  |  |  |  |  |  |  |
| *Linear-by-linear association between age categories | | | | | | | | | | | | | | |

Supplementary Table S3. Logistic regression analyzing the odds of venous thromboembolism within one year before the diagnosis of pancreatic cancer among pancreatic cancer patients diagnosed in 2013-2016 in Finland (n=4086)

|  |  | 95% CI | 95% CI |  |
| --- | --- | --- | --- | --- |
| Variable | Odds ratio | lower | higher | *p* |
| Radical surgery | 0.18 | 0.057 | 0.569 | 0.004 |
| Female (vs male) | 1.34 | 0.963 | 1.865 | 0.082 |
| Age at diagnosis | 0.968 | 0.953 | 0.984 | <0.001 |
| CCI 1 (vs 0) | 0.919 | 0.58 | 1.456 | 0.718 |
| CCI 2 | 1.294 | 0.806 | 2.076 | 0.285 |
| CCI 3+ | 1.812 | 1.179 | 2.783 | 0.007 |

CCI = Charlson comorbidity index score sum

Supplementary Results S4. Among cases excluded due to a post-mortem PC diagnosis [only death certificate (n = 172) or autopsy (n = 228)], VTE was the underlying cause of death in two (0.5%) cases, an immediate cause of death in 26 (6.5%) cases, and a contributing condition in an additional 7 (1.8%) cases.
